# Humic Substances as a Feed Supplement and the Benefits of Produced Chicken Meat

**DOI:** 10.3390/life13040927

**Published:** 2023-04-01

**Authors:** Slavomír Marcinčák, Boris Semjon, Dana Marcinčáková, Anna Reitznerová, Dagmar Mudroňová, Janka Vašková, Jozef Nagy

**Affiliations:** 1 Department of Food Hygiene, Technology and Safety, University of Veterinary Medicine and Pharmacy in Košice, Komenského 73, 041 81 Košice, Slovakia; 2 Department of Pharmacology and Toxicology, University of Veterinary Medicine and Pharmacy in Košice, Komenského 73, 041 81 Košice, Slovakia; 3 Department of Microbiology and Immunology, University of Veterinary Medicine and Pharmacy in Košice, Komenského 73, 041 81 Košice, Slovakia; 4 Department of Medical and Clinical Biochemistry, Faculty of Medicine, Pavol Jozef Šafárik University in Košice, 040 11 Košice, Slovakia

**Keywords:** broiler chicken, meat quality, humic substances

## Abstract

Humic substances with a high proportion of humic acids (more than 40%) have been classified by the European Commission as feed materials that can be used in animal nutrition since 2013. A protective effect on the intestinal mucosa, as well as anti-inflammatory, adsorptive and antimicrobial properties, were recorded. Nutrient absorption, nutritional status and the immune response in chickens supplemented with HSs were significantly improved. HSs have the ability to enhance protein digestion as well as the utilization of calcium and trace elements. They are known to improve feed digestibility as a result of maintaining an optimal pH in the gut, leading to lower levels of nitrogen excretion and less odor in the husbandry environment. HSs not only increase digestibility and result in greater utilization of the feed ration but also improve the overall quality of the meat produced. They increase the protein content and reduce the fat content in breast muscles. They also contribute to improving the sensory characteristics of the meat produced. Their antioxidant properties improve the oxidative stability of meat during storage. The influence of HSs on fatty acid composition may be one of the reasons that meat has a more beneficial effect on the health of consumers.

## 1. Introduction

Humic substances (HSs) are natural organic compounds found in soil, coal, water and other sources. They are formed by the biological and chemical decomposition of plant biomass via the activity of microorganisms. Their heterogenous macromolecular structures and compositions may vary depending on the site of occurrence.

According to their solubility, HSs are divided into humic acids, fulvic acids and humins. Humin has a high molecular weight and is insoluble in water regardless of the pH. Humic acids have a medium molecular weight and are insoluble in acidic environments with a pH less than 2; however, they become soluble in alkaline environments. Fulvic acids are soluble regardless of the pH of the environment and have the lowest molecular weight [1]. As the molecular weight increases, the carbon and oxygen contents, acidity and degree of polymerization also change [2]. 

The chemical structures of HSs are not fully known; they contain different functional groups (carboxylic, phenolic, carbonyl, hydroxyl, amine, amide and aliphatic). Due to their diverse molecular structures, many benefits in agriculture have been proven. They aid in the transport of micronutrients from the soil into plants, increase water retention and stimulate the growth of positive microorganisms in the soil. The ability of HSs to form chelate complexes with micronutrients and facilitate nutrient uptake by plants is also used in plant breeding [3].

Due to the diverse contents of functional groups, HSs, along with other natural substances, are among the most potent chelating agents. Compared to other inorganic adsorbents, such as zeolites, their adsorption capacity is several times higher. The ability of HSs to bind heavy metals, such as cadmium and lead, also increases with increasing atomic weight [4,5]. They are good adsorbents of heterogenous substances, which can eliminate or reduce the toxicity of endogenous or exogenous toxins.

Several studies have confirmed the effectiveness of HSs in reducing toxicity caused by aflatoxins [4,6]. Naturally occurring mycotoxins in contaminated feed are known to significantly impair animal growth parameters, organ morphology and the values of most blood biochemical parameters; they do not, however, cause clinical signs during short periods of feeding. HSs added to feed with low levels of mycotoxins act as adsorbents and thus modify the values of growth and biochemical parameters. Fulvic acids present in HSs form complexes with minerals and change their electrical charges, thus facilitating their faster uptake into the body. HSs induce an increase in the permeability of cell membranes and consequently facilitate the transport of minerals from the blood into cells [1].

## 2. The Use of HSs in Broiler Fattening

In the past, antimicrobials have been used as growth promoters in livestock nutrition. Due to the risk of drug residues and the increase in the resistance of microorganisms to antibiotics used in both veterinary and human therapeutics, their use in animal nutrition has been banned. Nowadays, HSs are considered to be one of several classes of suitable alternatives, and many positive effects on production parameters, the immune system and animal health have been attributed to them. They are able to bind various toxic substances and form insoluble complexes with them. Due to this property, they are also suitable for use as adsorbents and consequently are able to reduce the absorption of various endotoxins, which is of paramount importance in the protection of animal and human health. HSs have antibacterial, antiviral and antimicrobial effects in animal husbandry, thus improving the economics and ecology of livestock production (mainly by increasing growth, reducing the cost per kilogram of gain and minimizing the risk of disease). Moreover, they are neither toxic nor teratogenic [7,8].

In 2013, according to Commission Regulation 68/2013 [9], leonardite as a source of humic substances was included in the catalogue of feed materials that can be used in animal nutrition in the EU. In this regulation, leonardite is defined as a naturally occurring mineral complex of phenolic hydrocarbons, also known as humate, which originates from the decomposition of organic matter over the course of millions of years. HSs, as organic mineral feed with a high proportion of humic acids (more than 40%), have been classified as feed supplements used in the EU. In horses, cattle, sheep, goats, pigs and poultry, HSs serve as treatments for diarrhea, dyspepsia and acute intoxications. They also show a marked tendency to inhibit pathogenic bacterial and microscopic fungal growth, and therefore may reduce mycotoxin levels. A protective effect on the intestinal mucosa as well as anti-inflammatory, adsorptive and antimicrobial properties have also been recorded. HSs improve gut health, nutrient absorption, nutritional status and the immune response in animals. They have the ability to improve protein digestion as well as the utilization of calcium and trace elements significantly. They are known to improve food digestibility due to their property of maintaining an optimal pH in the intestines, resulting in lower levels of nitrogen excretion and less odor in the husbandry environment. Humic acids not only increase the digestibility and utilization of food, but they also improve the overall environment in the gastrointestinal tract [1].

## 3. Effects of Humic Substances on Growth Parameters and Feed Conversion

Additives of natural origin can be added to feed for the purpose of improving growth parameters, animal health and/or improving the quality of the meat produced [10]. In 1999, the EMEA (The European Agency for the Evaluation of Medicinal Products) issued the approval of the oral administration of humic acids to all food-producing animals. In animal production, the addition of humic acids to feed can positively influence all production parameters. Humates included in the feed or water of poultry promote their growth [11]. The positive effects of HSs added to the feed and water of broiler chickens at different concentrations on growth parameters (chick weight, gain, feed consumption and feed conversion) has been reported by several authors [7,12,13,14]. These effects ensure, among other things, the proper composition of the intestinal microflora [15,16]. The presence of organic acids suppresses the production of toxic products by bacteria and prevents the colonization of the intestine by pathogenic microorganisms [17]. HSs support the formation of a protective layer of the intestinal mucosa against pathogens and toxic substances that could cause reductions in weight gain and thus the final weight of chickens [18]. Feed conversion is also an important parameter that is monitored during fattening of chickens. Its value is calculated based on weight gain and feed consumption during the fattening period. Ozturk et al. [12] stated that the improvement of feed conversion and feed increments in poultry fed with the addition of 1.5 g of HSs per kg of compound feed proves their utilization as a suitable alternative to antimicrobial feed additives used as growth promoters, which was, furthermore, confirmed by other authors as well [11]. Arif et al. [18] found that the addition of HSs at 0.75, 1.5 and 2.25 g·kg^−1^ of feed resulted in an increase in final weight, feed intake and weight gain and also improved feed conversion values in quails. They noted that, as the concentration of HSs in feed increased, the final weight of chicks increased, feed intake decreased and feed conversion improved. A pronounced effect on growth parameters with an increasing concentration of HSs in feed was also observed by Jaďuttová et al. [14], where after feeding higher amounts of HSs in broiler feed (0.8 and 1.0%) there was a slight increase in final chick weight and gain, and, moreover, the differences in values were balanced. This finding is in agreement with the results of Eren et al. [19], who reported that feeding feed supplemented with HSs in a concentration of 2.5 g·kg^−1^ of compound feed significantly improved chick gains and feed conversion. Hudák et al. [20] added HSs to broiler chickens’ diets in natural and acidified forms at a 0.7% concentration. The acidified form contains formic acid that functions to increase feed digestibility. They noted that both forms of HSs had an effect on improving the final weight and achieving higher gains during fattening as well as better feed conversion compared to the control group. However, it is important to note that the acidified form had no effect on the growth parameters of chicks compared to the natural form of HSs. The effect of HS administration in drinking water on the final weight and weight gain was described by Lala et al. [7]. As the concentration of HSs in the water fed to chickens increased, their final weight and gains also increased. Similarly, feed conversion was better in chickens supplemented with HSs in water.

A positive finding for poultry farmers is that, after the addition of HSs to chick feed, feed conversion was improved and chickens achieved higher final weights, although statistical differences in final weight were not always noted. However, for farmers, an increase in chicken weight of 70–90 g per chicken on average is a significant economic benefit, which may represent a considerable economic benefit when the number of chickens reared per pen is large. 

On the contrary, no effect of HSs on growth parameters has been recorded [21]. Kaya and Tuncer [22] reported no improvement in feed conversion with the implementation of HSs in feed at a rate of only 0.25%. For the action of HSs to effectively improve growth parameters, the concentrations used are of chief importance. Regarding the above-mentioned experiments, the optimum dose of HSs was 0.5 to 1.0%. The exact concentration is always dependent, also, on the humic acid content, which should be at least 40%, and on the way the HSs are treated before use. 

## 4. Effect of Humic Substances on Carcass Yield

An important indicator of the efficiency of fattening and rearing poultry is the carcass yield, as is the yield of individual body parts of chickens. A positive effect of HSs on carcass yield was observed at concentrations ranging from 0.25 to 1.0%. The addition of 1% of HSs to chicken feed will ensure better carcass yield. A significant increase in breast and thigh muscle yield was observed in chickens fed with 1% HSs supplemented in broiler feed compared to the control group [14]. Feeding 0.75% HSs increased carcass yield as well. Breast muscle yield was comparable to that of the control group [20]. The addition of 0.6% HSs to chicken feed during a fattening period of 39 days had a significant effect on body yield, with higher body weights, as well as breast and thigh muscle weights, recorded [23]. These claims are in agreement with the work of Celik et al. [24], who reported significant increases in carcass and breast muscle yields of poultry after the addition of HSs at concentrations as low as 0.25%, and also with the results of Arif et al. [18], who confirmed the best values of carcass and breast muscle yields after feeding HSs at 2.25 g·kg^−1^ of the feed mixture.

## 5. Effect of Humic Substances on the Digestive Tract

The digestive tract of chicks is immature and sterile shortly after hatching. Chickens are very susceptible to pathogenic microorganisms until their gut microbiome develops. Antimicrobial feed additives have often been administered to suppress pathogenic microorganisms and to improve growth and fattening efficiency. The benefits of antibiotic administrations as promoters of animal health and growth have been well documented in the scientific literature. Unfortunately, the risks of pathogen resistance to antibiotics posing a serious threat to animal and human health have been equally well described [7].

For this reason, various alternatives, such as probiotics, prebiotics, plant-based ingredients and organic acids, have started to be used in poultry farming. Most of these alternative additives work by affecting the gut microbiome and the digestive process. HSs, representing one of these alternatives, have been said to inhibit the growth of bacteria and microscopic fungi and thus reduce mycotoxin levels in feed [25].

Acidification of the digestive tract by various organic acids reduces the formation of toxic bacterial products and colonization of the intestinal wall by pathogens, thereby preventing damage to the epithelial cells of the intestines [26]. Mudroňová et al. [15] investigated the effect of HSs on the microbiome of the small intestine and appendix; in addition, the contents of lactic acid bacteria and enterobacteria were also monitored. They noted that HSs at a 0.8% concentration in chicken feed had a positive effect on the gut microbiome, represented by a decrease in *Enterobacteriaceae* and, conversely, a significant increase in lactic acid bacteria, compared to the control group. A positive effect of HAs on the composition of the gut microbiome was also observed by Arif et al. [18]. They stated that, after the addition of 0.25 g·kg^−1^ of HSs, the contents of coliform bacteria, *E. coli* as well as *Clostridium perfringens* decreased in the ceca of quails. They also noted a decrease in the pH of the environment in the cecum.

In broilers, benefits of HS addition, such as increase in the length of the villi of the small intestinal mucosa and reduction in the depth of the villi due to the formation of a protective HS layer, have been reported [27,28]. 

Due to colloidal characteristics and a high capacity of HSs to form aggregates within solutions, it has been proposed that HSs have the ability to create protective layers on the epithelial mucous membrane of the digestive tract, preventing the penetration of pathogenic bacteria or toxic substances produced by bacteria [29] and improving the utilization of nutrients from feed [1]. HSs also interact with biomolecules, such as collagen, promoting the resistance and maturity of its fibers, resulting in an increase in intestinal villi integrity [29,30]. Ceylan and Ciftci [31] state that HSs can increase the uptake of nitrogen, phosphorus and other nutrients due to their chelating properties. The acids’ anions bind with calcium, phosphorus, magnesium and zinc, resulting in the improved digestibility of these minerals, which serve as substrates for intermediary cell metabolism [7].

## 6. Influence on the Immune System

HSs also exert a beneficial effect on the immune system of poultry. Nagaraju et al. [21] noted an improvement in the production parameters and the immune status of broilers after the addition of HSs to antibiotic-free feed. There are several ways in which substances can influence the immune system. One of the modes of action is the formation of solid complexes of HSs with carbohydrates which subsequently enable the formation of glycoproteins capable of binding to NK cells and T lymphocytes [29,32]. These glycoproteins act as modulators of intercellular communication and therefore regulate the immune response, including regulation of cytokine production and preventing excessive activity of cytotoxic T lymphocytes and natural killer cells. Subsequently, cytokines affect/regulate a number of immune reactions in the organism [32,33]. 

In several experiments, an effect on the representation of poultry lymphocytes was noted. An increase in total lymphocyte numbers after HS application was noted in laying hens [34], broilers [35] and Japanese quails [36]. Cetin et al. [34] reported that feeding humic acids (at 0.15%) to poultry resulted in a significant increase in the number of lymphocytes via increased IL-2 production and expression of IL-2 receptors on lymphocytes, which led to an increase in the IL-2 production activity of the cells. In addition, changes in the representation of individual lymphocyte subpopulations were also observed. After feeding 0.8% HSs from leonardite to broiler chickens, there was an increase in the representation of T lymphocytes—specifically, helper T lymphocytes—whereas cytotoxic T lymphocytes were reduced. The gene expression of IgA was not changed [15]. In contrast, in laying hens receiving 0.5% HSs, an increased percentage of the B lymphocyte subpopulation was noted, which corresponds to the increased gene expression of IgA in the intestines. The expression of genes for mucin production (MUC-2) was also increased, which, together with IgA, is significantly involved in the protection of mucosal surfaces, thereby improving feed utilization [16]. Rath et al. [35] found that, after feeding HSs at 0.25%, there was an increase in the weight of the bursa of Fabricius—a key organ for the development and differentiation of B lymphocytes in birds. The activation of B lymphocytes was confirmed, also, by significant increase in serum IgM and IgG levels in laying hens after 0.1 and 0.5% HS employment [37] and by increased serum gamma globulins in broilers [38]. 

Another mechanism which is strongly influenced by HSs is phagocytosis. In our previous experiments during which broilers received 0.8% and laying hens 0.5% HSs in feed, we noted significantly higher values of active phagocytes as well as their engulfing capacity [15,16]. Similar results were observed, also, in other animal species [39]. Sanmiguel and Rondón [40] found that the effect of HSs on phagocytes depends on time. The addition of 0.1 and 0.2% HSs to laying hens’ diets was associated with stimulated phagocytosis after 8 and 30 days of application; however, phagocytic activity was significantly reduced after 60 days as compared to the control group. Similarly, they also noted an increase in the oxidative burst of phagocytes at day 30 and a decrease at day 60. It is not completely clear how HSs act on phagocytosis, but it has been confirmed that HSs stimulate the adhesion abilities of phagocytes and the production of reactive oxidative intermediates and that they are able to induce nuclear factor κB, which is decisive for the transcription of many genes involved in the process of phagocytosis (e.g., GMCSF, IL-8 and TNF-α) [41].

Based on the results of scientific studies, it can be assumed that the effect of humic substances on the immune system is affected by the concentration and duration of application and by the category and species of chickens to which the HSs are administered. These facts should be taken into account when applying HSs on farms in order to achieve an optimal effect.

## 7. Bone Mineralization

Calcium and phosphorus are macronutrients that are essential for bone formation. Insufficient dietary calcium sources can lead to hypocalcemia in the blood, which may lead to decreased bone strength and mineralization. HSs are considered to be excellent natural sources of minerals, as they have a high complexation capacity and are able to form chelates with different ions [29]. Their application is therefore associated with improved mineral utilization by plants and animals [2]. Angeles et al. [42] investigated the effect of HSs in water on the calcium and phosphorus contents of tibia bones. Chickens were administered HSs at concentrations of 161, 322, 483 and 644 µg·L^−1^. They noted that increasing concentrations of HSs in water resulted in increased tibial Ca levels and improved bone mineralization in broiler chickens at 21 and 42 days of age. The effect of HSs (0.8 and 1.0% supplementation in feed mixture) on bone mineral composition was also evaluated by Jaďuttová et al. [14]. They observed a significant increase in the amount of calcium and a decrease in the amount of phosphorus in the long bones (tibias) of broilers, with a decrease in the amount of these macronutrients in the blood. They also noted better mineralization as well as bone quality. A possible explanation of the lower amount of calcium in the blood of the chickens could be that there was a higher accumulation of calcium in the bones of the experimental groups fed with HSs. The high accumulation of calcium in bones and the pronounced growth rate of broiler chickens may have caused a drop in calcium blood levels at the time of slaughter. Similar findings are also presented in the works of Rath et al. [35] and Ozturk et al. [43], in which reductions in the serum concentrations of calcium, magnesium and phosphorus in the blood of broiler chickens were also recorded. A lower concentration of calcium and phosphorus in blood serum may be due to the ability of HSs to chelate metals, which is influenced by the large number of carboxylic acid side chains [2,35] that they have.

## 8. Humic Substances and Meat Quality of Broiler Chickens

Poultry meat is popular among consumers due to its high protein content, low fat content and its being a source of vitamins and minerals. The quality of meat can vary depending on its chemical composition, which is influenced by the diet and substances added to the animal feed. HSs have been tested quite intensively as feed additives in recent years. However, their effects are mainly studied in regard to growth parameters as replacements for antibiotic growth promoters, improvement of the health status of broiler chickens and reduction in the use of antibiotics for the treatment of chickens [12]. In terms of meat quality, there are fewer records regarding the effects of humic substances [20,44,45,46,47]. Humic substances administered to broiler chickens influenced the basic chemical composition of the meat. In the conducted experiments, a decrease in fat content and an increase in protein content in breast muscles was observed, along with an increase in fat content and a slight decrease in protein content in the thigh muscles of chickens [12]. According to Wang et al. [48], the decrease in fat thickness and increase in the marbling of the meat produced after feeding humic ingredients suggests that humic ingredients are capable of influencing the distribution of fats and proteins in the body and thus altering the composition of the meat. Semjon et al. [47] and Hudák et al. [20] reported that after feeding humic substances there is a decrease in fat content and an increase in protein content in breast muscles. However, Semjon et al. [47] reported a significantly higher fat content in chicken thigh muscles after the addition of 0.8% HSs. Similarly, Ozturk et al. [12] reported that the addition of 0.5, 1.0 and 1.5% concentrations of HSs affected the fat and total protein contents differently. While the total protein content decreased after feeding feed supplemented with 1.0% HSs, no significant effect on the total protein content of breast muscles after feeding 0.5 and 1.5% concentrations was observed. On the contrary, all three experimental groups showed decline in the breast meat fat content compared to the control group. The results indicate that diets with HS addition cause reduction in fat content and, conversely, increase in the protein content of chicken breast muscles. This finding is very encouraging, especially regarding human dietary recommendations, where a lower fat diet and a higher protein diet is preferred. Meat of broiler chickens fed with feed supplemented with HSs can be considered a very valuable meat type compared to conventional commercial meat due to its improved nutritional composition.

Several indicators of meat quality are known. One of the main parameters of meat quality is pH [49]. Levels of 5.8 or less 24 h after slaughter are recommended; higher values cause changes in meat quality, especially regarding color and tenderness [50,51]. Semjon et al. [47] reported a lower pH in the breast and thigh muscles of broiler chickens after the addition of 0.8% and 1.0% humic substances in diets. Similarly, Hudák et al. [20] also reported a decrease in the pH of thigh muscles after the addition of 0.7% HSs in the diet of broiler chickens. In comparison, Akaichi et al. [52] observed no effect on the pH of chicken breast muscles after feeding humic acids (0.1%) in feed. Similarly, Ozturk et al. [12] did not report changes in the pH of chicken breast muscles after feeding 0.5–1.5 g·kg^−1^ HSs in feed. In the thigh muscles, the pH of the meat was slightly higher compared to the control group. However, it is of importance that more experiments need to be conducted to unambiguously determine the effect of HSs on meat pH.

Meat color is an important factor for the market. Meat color can be evaluated instrumentally or by a sensory panel. The instrumental approach is based mainly on the CIE system (International Commission on Illumination) that functions as the standard for color specification and measurement universally accepted. It takes three fundamental aspects into consideration, namely, luminosity or lightness (L*), red tones or redness (a*), and blue–yellow tones or yellowness (b*) [53]. The use of humic substances causes a change in the color of the breast muscles. However, the concentration of HSs used in feed plays an important role regarding the color of the meat. Hudák et al. [20] reported significantly lighter breast muscle meat after feeding 0.7% HSs in acidified forms. Akaichi et al. [52] did not observe any changes in the color of the breast muscles of chickens after feeding HSs compared to the control group. Disethle et al. [54] reported similar results. Semjon et al. [47] noted a change in the lightness and redness of breast meat with a 1.0% supplementation of HSs. The meat was observed to be significantly darker and redder in color than the meat of chickens from the control group. Even though the results on the effect of HSs on meat color are not unambiguous, we can conclude that HS feeding has a beneficial rather than a negative effect and is dependent on the concentration of HSs used in the feed.

An important parameter for the acceptance of meat as an important food is its sensory qualities. Feed ingredients that can influence the sensory evaluation of meat are also of interest to poultry farmers and meat producers, as they can increase the attractiveness of products to consumers. HSs positively affect the sensory quality of meat. Semjon et al. [47] noted in the sensory evaluation of chicken breast meat a positive response with regard to the perception of meat quality in relation to the supplementation of humic substances in the diet. In particular, they noted a significant improvement in meat flavor after feeding 1.0% HSs. Sensory evaluation of taste indicated a positive response with respect to the perception of meat quality in relation to increased supplementation of HSs in the diet. A beneficial effect of HSs on the sensory evaluation of breast muscles of chickens was also reported by Akaichi et al. [52]. They noted a significant improvement in meat color and aroma. Čurlej et al. [46] noted an improvement in meat texture after the addition of 0.6% HSs to chicken feed. These results also confirm the beneficial effect of HSs in improving the sensory quality of meat and thus increasing its attractiveness to consumers.

Beneficial effects of adding HSs to chicken feed regarding the water-binding capacity of meat and the water loss of meat after cooking were also observed. HSs had the effect of increasing the water-holding capacity of both breast and thigh muscles [12]. Another interesting finding from a consumer perspective is the increased water-holding capacity of meat after heat treatment [47]. This does not significantly reduce the weight of the meat and also preserves the juiciness of the meat. This may also be the reason why the meat of HS-supplemented chickens is evaluated as better from the sensory aspect after HS feeding.

## 9. Effect of HSs on the Fatty Acid Profile of Meat Fat

The effect of HSs on fatty acid profiles has not been investigated so far. Therefore, our research is focused on the effect of adding humic substances to broiler chicken feed mixtures on the fatty acid composition of meat and cavity fat. A significant finding is that, after feeding humic substances at concentrations of 0.8 and 1.0%, the n-6/n-3 PUFA ratio in the breast muscles decreased. There was also a significant increase in the proportion of oleic acid and a decrease in the proportion of arachidonic acid compared to the control group’s breast muscles (Table 1). When fed 0.6% HSs, the n-6/n-3 PUFA ratio remained unchanged in both breast and thigh muscles (Table 2). However, there was a decrease in the proportion of oleic acid and an increase in arachidonic acid in the thigh muscles. We observed a desirable increase in the proportion of n-3 PUFAs, DPA, EPA and DHA after feeding with all three tested concentrations. The body fat profile was minimally affected by a decrease (0.6%) or increase (0.8 and 1.0%) in the proportion of oleic acid. In general, it can be concluded that humic substances have a different effect on the fatty acid profile of meat fat (breast and thigh muscles) and a different effect on body fat. A more pronounced effect of feeding humic substances on the fatty acid composition of meat fat was observed. The fatty acid profile of body fat was affected minimally and rather adversely. We can also conclude that humic substances, as natural substances, are able to reduce the proportion of n-6/n-3 PUFAs in meat fat. This is mainly due to a reduction in the proportion of arachidonic acid and a slight increase in EPA, DPA and DHA in the fat of chicken meat. Interestingly, different HS concentrations in broiler feed had different effects on the fatty acid profile. However, we cannot state unequivocally that the proportions of certain acids increased or decreased with increasing concentrations of humic components in chicken feed. Further studies, especially in terms of fat quality, will be needed in the future to confirm unequivocally the influence of different concentrations of humic substances on the fatty acid profile of the meat produced.

## 10. Oxidative Changes in Lipids after Humic Acid Supplementation

Lipid oxidation affects the most important quality attributes, including sensory (flavor, color and texture) and functional properties (water-holding capacity and emulsifying ability), of meat. Therefore, the prevention of lipid oxidation in meat is important for meat quality and also for human health [55]. Humic acids are active substances with antioxidant effects—they promote the activity of antioxidant enzymes [56]. Vašková et al. [57] indicate strong antioxidant effects of humic acids in the body by the promotion of antioxidant enzymes. Aeschbacher et al. [58] report that the antioxidant effects may be based on the phenolic and quinone groups present in the structures of humic substances, which are electron donors and acceptors, providing their antioxidant effects. Polyphenols—lignin-derived compounds—are considered to be some of the main components of humic substances that contribute to their antioxidant effects [59]. In addition, they are able to chelate metals, especially iron and copper, inhibiting the formation of free radicals via transition metal catalysis, thereby controlling lipid peroxidation and DNA fragmentation [60]. Their antioxidant effects, although not precisely described, can also be seen in the stabilization of meat fats. The addition of 0.7% humic substances can ensure decreased lipid oxidation and increased antioxidant activity of broiler meat during chilled storage conditions [20]. Aksu et al. [44] observed a decrease in lipid oxidation in vacuum-packed breast and thigh muscles during chilled storage after the use of 0.1, 0.2 and 0.3% commercial humate in broiler chicken diets.

Reitznerová et al. [61] reported that the oxidative stability of poultry meat after humic substance feeding was favorably affected and that breast meat stored for 12 months in the freezer had higher oxidative stability compared to that of the control group. Similarly, Marcinčáková et al. [62] reported that, after feeding humic substances at a dose of 0.8% to chickens in the feed, the antioxidative stability of meat stored in a refrigerator and meat frozen for 12 months was similar to the antioxidative activity of meat from the control group. The results of Semjon et al. [47] also confirmed that humic substances in the diet of broiler chickens did not negatively affect nor increase the oxidative stability of the meat produced. The oxidative stability of meat after feeding 0.8 and 1.0% humic substances in the diet was not affected by the humic substances. On the contrary, the addition of humic substances slightly improved the oxidative stability of meat stored for 7 days in the refrigerator (Table 3).

The better oxidative stability of the meat of broilers fed with HS addition could have been caused by a lower content of fat in comparison to meat of the control group, as noted also by Ozturk et al. [12]. The fatty acid profile of the meat after humic acid digestion did not change significantly, and, interestingly, there was an increase in oleic acid, which is oxidatively stable. Oxidation of thigh muscle fat is higher during storage, as thigh meat contains more fat and also a slightly higher proportion of unsaturated fatty acids than breast muscle meat [61]. However, the effect of HSs, as antioxidant components, was higher in thigh muscle meat, which was reflected in the lower amounts of oxidation products compared to the control group. This also supports the observation that, as the proportions of monounsaturated and polyunsaturated fatty acids in broiler chicken meat increased, fat oxidation also increased during storage of the samples [63]. Although the antioxidant effect of HSs in living organisms has been demonstrated, the effect of HSs on oxidative changes in meat has not been clearly confirmed. Recent work carried out suggests that HSs have an effect on the oxidative stability of fats at higher concentrations (1%) and in meat with a higher fat content.

## 11. Conclusions

The present review presents information on the beneficial effects of HSs in the diet of broiler chickens. The main confirmed benefits of using HSs as feed materials from the perspective of broiler chicken breeding are the formation of protective barriers on the intestinal mucosa, the stabilization of the intestinal microflora and the improvement of the immune system of broiler chickens. Equally important benefits of HS feeding include the improved bone quality of chickens, improved feed conversion and, in part, the final weight of chickens. 

In terms of the quality of the meat produced, an important benefit of HS feeding is the increase in protein content and the decrease in fat content of the breast meat produced. Positive findings also include the effect on the sensory characteristics of the meat, such as improved color and flavor. Among the confirmed effects of HSs on meat quality are the proven higher water-binding capacity of meat and thus lower water loss in meat after cooking. The addition of HSs can ensure lower lipid oxidation and higher antioxidant activity of broiler meat during chilling and freezing storage. A beneficial effect of HSs was also observed on the fatty acid profile of chicken breast muscle fat. However, at present, there are fewer data in this area and more experiments need to be carried out. 

Although there are many scientific papers confirming the beneficial effects of HSs on the health and production parameters of broiler chickens, as well as on meat quality, their mechanism of action remains unclear. However, their use in broiler chicken fattening is, according to scientific experiments, the right choice to improve the health and productivity of broiler chickens, as well as to increase the quality of the meat produced.

## Figures and Tables

**Table 1:** The effect of HS feed addition on the fatty acid profiles of broilers’ breast meat samples (means ± SDs).

Fatty Acid	C	HS0.6	HS1.0
C 16:0	21.62 ± 0.12 ^a^	21.38 ± 0.14 ^a^	19.44 ± 0.96 ^b^
C 16:1 n-9	4.02 ± 0.53	4.13 ± 0.06	3.81 ± 0.39
C 18:0	9.43 ± 0.26	9.72 ± 0.18	10.14 ± 1.18
C 18:1 n-9	30.13 ± 0.52 ^b^	33.04 ± 0.52 ^a^	32.30 ± 0.82 ^a^
C18:1 n-7	3.89 ± 0.28	3.75 ± 0.17	3.97 ± 0.32
C18:2 n-6	16.34 ± 0.13 ^a^	15.40 ± 0.17 ^b^	15.46 ± 0.40 ^b^
C18:3 n-6	0.119 ± 0.01	0.12 ± 0.02	0.167 ± 0.05
C18:3 n-3	0.82 ± 0.05	0.95 ± 0.07	0.883 ± 0.08
C 20:2	0.49 ± 0.078	0.63 ± 0.06	0.576 ± 0.14
C20:3	0.08 ± 0.02 ^b^	0.16 ± 0.02 ^a^	0.10 ± 0.02 ^b^
C20:3 n-6	1.20 ± 0.05	1.07 ± 0.06	1.10 ± 0.06
C20:4 n-6	5.75 ± 0.03 ^a^	4.07 ± 0.02 ^b^	5.05 ± 0.02 ^b^
C20:5 n-3	0.37 ± 0.05	0.35 ± 0.02	0.39 ± 0.02
C22:6 n-3	0.66 ± 0.02	0.51 ± 0.03	0.55 ± 0.03
∑SFAs	34.42 ± 0.39	35.28 ± 1.02	33.16 ± 1.43
∑UFAs	65.58 ± 0.43	64.72 ± 1.04	66.84 ± 1.43
∑PUFAs n-3	1.93 ± 0.07	2.06 ± 0.13	2.16 ± 0.11
∑PUFAs n-6	23.89 ± 0.19 ^a^	21.46 ± 0.10 ^c^	22.49 ± 0.10 ^b^
n-6/n-3	12.41 ± 0.38 ^a^	10.49 ± 0.20 ^b^	10.41 ± 0.22 ^b^

C—control group with standard diet; 0.6% HS—0.6% addition of HSs to feed; 1.0% HS—1.0% addition of HSs to feed; C 18:1 n-9—oleic acid; C18:1 n-7—vaccenic acid; C18:2 n-6—linolenic acid; C18:3 n-6—gamma-linolenic acid; C18:3 n-3—alfa-linolenic acid; C20:3 n-6—dihomo-gamma-linolenic acid; C20:4 n-6—arachidonic acid; C20:5 n-3—eicosapentaenic acid; C22:6 n-3—docosahexaenic acid; SFAs—saturated fatty acids; UFAs—unsaturated fatty acids; PUFAs—polyunsaturated fatty acids. Values in rows with a different mark (^a–c^) are significantly different (*p* < 0.05).

**Table 2:** The effect of HS supplementation on the fatty acid profiles of broilers’ breast meat samples (means ± SDs).

Fatty Acid	C	HS0.6
C14:0	1.92 ± 0.04	2.15 ± 0.37
C16:0	20.76 ± 0.18	20.66 ± 0.24
C16:1 n-9	2.64 ± 0.07 ^b^	4.26 ± 0.17 ^a^
C18:0	10.99 ± 0.12 ^a^	9.65 ± 0.18 ^b^
C18:1 n-9	24.80 ± 0.11	25.41 ± 0.39
C18:2 n-6	17.74 ± 0.15 ^b^	18.55 ± 0.19 ^a^
C18:3 n-6	0.07± 0.01	0.08 ± 0.01
C18:3 n-3	0.69 ± 0.01	0.76 ± 0.12
C20:2 n-6	0.89 ± 0.02	0.84 ± 0.05
C20:3 n-3	0.10 ± 0.01	0.12 ± 0.02
C20:3 n-6	1.62 ± 0.02 ^b^	1.54 ± 0.04 ^a^
C20:4-n-6	7.17 ± 0.04 ^a^	4.73 ± 0.32 ^b^
C20:5 n-3	0.70 ± 0.02 ^a^	0.44 ± 0.11 ^b^
C22:5 n-3	1.36 ± 0.03 ^a^	1.20 ± 0.04 ^b^
C22:6 n-3	1.27 ± 0.01 ^a^	1.15 ± 0.03 ^b^
∑SFAs	35.44 ± 0.21	35.14 ± 0.83
∑UFAs	64.56 ± 0.21	64.86 ± 0.83
∑PUFAs n-3	4.16 ± 0.03 ^a^	3.71 ± 0.08 ^b^
∑PUFAs n-6	26.61 ± 0.17 ^a^	24.90 ± 0.33 ^a^
n-6/n-3	6.40 ± 0.26	6.72 ± 0.16

C—control group; 0.6% HS—0.6% addition of HSs to broilers’ diet; C14:0—myristic acid; C18:1 n-9—oleic acid; C18:2 n-6—linolenic acid; C18:3 n-6—gamma-linolenic acid; C18:3 n-3—al-fa-linolenic acid; C20:3 n-6—dihomo-gamma-linolenic acid; C20:4-n-6—arachidonic acid; C20:5 n-3—eicosapentaenic acid; C22:5 n-3—docosapentaenic acid; C22:6 n-3—docosahexaenic acid; SFAs—saturated fatty acids; UFAs—unsaturated fatty acids; PUFAs—polyunsaturated fatty acids. Values in rows with a different mark (^a^,^b^) are significantly different (*p* < 0.05).

**Table 3:** The oxidative stability of broilers’ breast meat fat during storage via chilling (4 °C, 7 days) and freezing (−18 °C, 365 days) conditions expressed as malondialdehyde content (mg·kg^−1^ means ± SDs).

Sample	Day 1	Day 7	Day 365
C	0.101 ± 0.016	0.129 ± 0.023	0.176 ± 0.027
HS0.8	0.132 ± 0.017	0.096 ± 0.011	0.197 ± 0.012
HS1.0	0.095 ± 0.005	0.099 ± 0.013	0.199 ± 0.034

C—control group; HS0.8—broilers’ diet supplemented with 0.8% of HSs; HS1.0—broilers’ diet supplemented with 1.0% of HSs.

## Data Availability

Not applicable.
